# Esporotricosis en Argentina: análisis clínico y epidemiológico

**DOI:** 10.7705/biomedica.6886

**Published:** 2023-08-31

**Authors:** Gabriela Santiso, Fernando Messina, Alicia Arechavala, Emmanuel Marín, María de las Mercedes Romero, María de los Ángeles Sosa, Florencia Rojas, Javier Mussin, Sonia Contreras, Viviana Galache, María Guerrero, Vanesa Sosa, Yone Chacón, Christian Álvarez, Ivana Maldonado, Mercedes Romero, Sofía Echazarreta, Norma Fernández, Silvia Relloso, Julián Serrano, Gustavo Giusiano

**Affiliations:** 1 Unidad de Micología, Hospital de Enfermedades Infecciosas Francisco Javier Muñiz, Buenos Aires, Argentina Unidad de Micología Hospital de Enfermedades Infecciosas Francisco Javier Muñiz Buenos Aires Argentina; 2 Laboratorio Central Corrientes, Instituto de Medicina Regional, Universidad Nacional del Nordeste, Corrientes, Argentina Universidad Nacional del Nordeste Laboratorio Central Corrientes Instituto de Medicina Regional Universidad Nacional del Nordeste Corrientes Argentina; 3 Instituto de Medicina Regional, Universidad Nacional del Nordeste, CONICET, Corrientes, Argentina Universidad Nacional del Nordeste Instituto de Medicina Regional Universidad Nacional del Nordeste CONICET Corrientes Argentina; 4 Hospital de Alta Complejidad El Calafate, SAMIC, Santa Cruz, Argentina Hospital de Alta Complejidad El Calafate SAMIC Santa Cruz Argentina; 5 Laboratorio de Alta Complejidad (LACMI), Hospital Madariaga, Posadas, Argentina Laboratorio de Alta Complejidad (LACMI) Hospital Madariaga Posadas Argentina; 6 Hospital Señor del Milagro, Salta, Argentina. Hospital Señor del Milagro Salta Argentina; 7 Facultad de Bioquímica, Química y Farmacia, Laboratorio de Salud Pública de Tucumán, Tucumán, Argentina Facultad de Bioquímica, Química y Farmacia Laboratorio de Salud Pública de Tucumán Tucumán Argentina; 8 Laboratorio de Microbiología, Hospital Alemán, Buenos Aires, Argentina Laboratorio de Microbiología Hospital Alemán Buenos Aires Argentina; 9 CEMAR, Departamento Bioquímico, Secretaría de Salud Pública, Rosario, Argentina CEMAR Departamento Bioquímico Secretaría de Salud Pública Rosario Argentina; 10 Sala 9, Hospital de Enfermedades Infecciosas Francisco Javier Muñiz, Buenos Aires, Argentina Sala 9 Hospital de Enfermedades Infecciosas Francisco Javier Muñiz Buenos Aires Argentina; 11 Laboratorio de Micología, Hospital de Clínicas, Buenos Aires, Argentina Laboratorio de Micología Hospital de Clínicas Buenos Aires Argentina; 12 Laboratorio de Microbiología, Centro de Educación Médica e Investigaciones Clínicas “Norberto Quirno”, Argentina Laboratorio de Microbiología Centro de Educación Médica e Investigaciones Clínicas “Norberto Quirno” Argentina; 13 Sección de Micología, Hospital Independencia, Santiago del Estero, Argentina Sección de Micología Hospital Independencia Santiago del Estero Argentina

**Keywords:** Sporothrix, esporotricosis, micosis, Argentina, Sporothrix, sporotrichosis, mycoses, Argentina

## Abstract

**Introducción.:**

La esporotricosis es una micosis de implantación causada por *Sporothrix* spp. Este se encuentra distribuido mundialmente y se puede encontrar en la vegetación y en el suelo. La ruta más frecuente de adquisición de la infección es por traumatismos con elementos contaminados con propágulos del hongo. Los gatos domésticos son los animales más afectados y pueden transmitirla a los humanos, por lo que es considerada una zoonosis. Las formas clínicas incluyen: la linfangítica nodular, la cutánea fija, la pulmonar (poco habitual) y la diseminada (excepcional).

**Objetivo.:**

Analizar la epidemiología de la esporotricosis en Argentina entre los años 2010 y 2022. Describir la presentación clínica, los métodos de diagnóstico y el tratamiento de los casos diagnosticados en este período. Conocer los genotipos circulantes y observar su relación con el lugar geográfico de adquisición de la infección.

**Materiales y métodos.:**

Se llevó a cabo un estudio analítico, retrospectivo y observacional, en el que se analizaron las historias clínicas de los pacientes con esporotricosis de 12 instituciones de salud de Argentina, entre los años 2010 y 2022.

**Resultados.:**

Se presentan 54 casos en los que la forma clínica más frecuente fue la linfangítica nodular y el tratamiento de elección fue el itraconazol. En todos los casos se realizó diagnóstico convencional. El cultivo de las muestras clínicas resultó más sensible que el examen directo, ya que permitió el desarrollo de *Sporothrix* spp. en los 54 casos. En 22 casos se hizo identificación molecular y *Sporothrix schenkii sensu stricto* fue la especie más frecuentemente aislada.

**Conclusiones.:**

Este estudio permitió conocer la epidemiología de esta micosis en Argentina, así como la disponibilidad de métodos diagnósticos y el tratamiento de elección.

## Abstract (en)

**Introduction.:**

Sporotrichosis is an implantation mycosis caused by *Sporothrix* spp. It is distributed worldwide and can be found in vegetation and soil. The most frequent route of infection is by trauma with elements contaminated with fungal propagules. Since domestic cats are the most affected animals and can transmit this infection to humans, sporotrichosis is considered a zoonosis. Clinical presentations include nodular lymphangitis, fixed cutaneous, pulmonary (rare), and disseminated (exceptional).

**Objectives.:**

To analyze the epidemiology of sporotrichosis in Argentina during 2010 and 2022. To describe the clinical presentation, diagnostic methods, and treatment of cases diagnosed during this period. To know the circulating genotypes and to observe possible associations with the geographic location where the infection was acquired.

**Materials and methods.:**

Analytical, retrospective, and observational study. We analyzed the medical records of patients with sporotrichosis from 12 health institutions in Argentina, between 2010 and 2022.

**Results.:**

We present 54 cases in which the most frequent clinical form was nodular lymphangitis, and the treatment of choice was itraconazole. Conventional diagnosis was made in all cases. Culture of clinical samples was more sensitive than direct examination because it allowed the isolation of *Sporothrix* spp. in all 54 cases. Molecular identification was performed in 22 cases, with *Sporothrix schenkii sensu stricto* being the most frequently isolated species.

**Conclusions.:**

This study allowed to know the epidemiology of this mycosis in Argentina, as well as the availability of diagnostic methods and the treatment of choice.

La esporotricosis es una micosis de implantación causada por *Sporothrix* spp., un hongo dimorfo de distribución mundial, que se encuentra en la vegetación, en materia orgánica en descomposición y en el suelo. Se describe como una sapronosis cuya forma más frecuente de transmisión es por traumatismo con elementos contaminados con propágulos del hongo ^1^.

Varios mamíferos son susceptibles a esta infección y los gatos domésticos son los animales que se ven afectados con mayor frecuencia ^2^. La esporotricosis felina se adquiere por arañazos o mordeduras, o por contacto directo con las secreciones de otros gatos, lo que puede provocar epizootias. Asimismo, estas mascotas pueden transmitirla a los humanos, lo que la convierte en una zoonosis. La ruta alternativa de infección está relacionada con la transmisión animal horizontal, lo que facilitaría la entrada directa de la fase levaduriforme del hongo al individuo ^2^^-^^5^. La inhalación de propágulos fúngicos (conidios o levaduras) puede ser la causa de las lesiones pulmonares o de las formas diseminadas ^5^^,^^6^.

Las infecciones por hongos dimorfos suelen observarse en zonas geográficamente limitadas. En particular, la esporotricosis tiene alta incidencia en Brasil, China y Sudáfrica ^7^. En el último tiempo, Brasil ha experimentado uno de los mayores brotes epidémicos de esporotricosis de origen zoonótico. Estos brotes se correlacionan con un incremento de casos de esporotricosis en gatos ^2^^-^^5^^,^^8^.

Entre las posibles presentaciones clínicas de esta micosis, la forma linfangítica nodular es la más frecuente, seguida por la forma cutánea fija. La forma pulmonar es poco habitual y la forma diseminada es excepcional y se da especialmente en huéspedes inmunocomprometidos ^2^^,^^6^.

Los gatos son los huéspedes animales más susceptibles a la infección por *Sporothrix* spp. y pueden desarrollar formas graves de esta micosis. La localización más frecuente de la infección es la región cefálica, principalmente la zona de la nariz. Su frecuencia es mayor entre los gatos machos adultos, sin dueño y sin castrar. En estos casos, el examen directo permite observar un elevado número de levaduras en las muestras obtenidas de estas lesiones ^2^^,^^7^^,^^9^, a diferencia de lo que ocurre con las lesiones en humanos.

Se dispone de escasos agentes antifúngicos efectivos para tratar la esporotricosis felina. Los casos de fracaso terapéutico son frecuentes en el tratamiento con itraconazol por lo que debería considerarse el uso combinado de distintos antifúngicos con efecto sinérgico ^10^. El éxito del tratamiento depende de múltiples factores, pero se asocia principalmente con la interacción huésped-hongo ^11^.

En la esporotricosis humana, el tratamiento de elección en las formas cutáneas o linfocutáneas es el itraconazol ^12^. También suele utilizarse el yoduro de potasio como primera alternativa ^12^. En las formas diseminadas o extracutáneas el tratamiento se realiza inicialmente con anfotericina B y luego se continúa con itraconazol ^6^.

La aplicación de técnicas moleculares para tipificar agentes patógenos fúngicos ha dado como resultado el reconocimiento de especies crípticas en varios géneros. La identificación definitiva de estas especies puede ayudar a definir el origen geográfico del evento causal, así como la fuente probable en los casos en que el paciente no la reconozca ^3^.

Durante más de un siglo, *Sporotrhrix schenckii sensu stricto* se describió como el único agente causal de esporotricosis humana y animal ^1^^,^^3^^,^^9^^,^^13^. Debido a los avances en la identificación molecular, actualmente se sabe que este género comprende aproximadamente 51 especies ^14^. Entre ellas, especies de interés clínico como *Sporothrix brasiliensis, Sporothrix schenckii, Sporothrix globosa* y *Sporothrix luriei*. El resto son especies ambientales y poseen poca o ninguna virulencia para los huéspedes vertebrados de sangre caliente ^2^^,^^3^. Los miembros del complejo *Sporothrix pallida (Sporothrix chilensis, Sporothrix gemella, Sporothrix humicola, Sporothrix mexicana, Sporothrix pallida, Sporothrix palmiculminata, Sporothrix protea-sedis* y *Sporothrix stylites*) presentan un leve potencial patógeno para animales y humanos ^3^^,^^7^.

Entre las pruebas de diagnóstico existen algunas basadas en la detección de antígenos y anticuerpos circulantes, pero el estudio micológico, que incluye el examen directo y el cultivo, son los más utilizados. Luego del aislamiento, la identificación de la especie puede realizarse por distintas técnicas moleculares, entre estas la espectrometría de masas o la amplificación y la secuenciación de genes específicos. La genotipificación a nivel de especie puede ayudar a definir el origen geográfico del evento responsable de esta micosis, así como la probable fuente cuando el paciente no la identifique ^2^.

Conocer la epidemiología de la esporotricosis permite generar políticas de prevención considerando las distintas fuentes de infección, sobre todo aquellas relacionadas con animales.

El objetivo de este estudio fue analizar la epidemiología de la esporotricosis en Argentina entre los años 2010 y 2022; asimismo, describir la presentación clínica, los métodos de diagnóstico y el tratamiento de los casos diagnosticados en este período, conocer los genotipos circulantes y observar su relación con el lugar geográfico de adquisición de la infección.

## Materiales y métodos

Se trata de un estudio analítico, retrospectivo y observacional. Se analizaron las historias clínicas de los pacientes con diagnóstico de esporotricosis registrados por 12 instituciones de salud de Argentina entre los años 2010 y 2022.

Como criterio de inclusión en esta casuística se consideraron aquellos pacientes con estudio micológico que incluían el examen directo en fresco y con coloración de Giemsa y cultivos del material obtenido de las lesiones. En los que se obtuvo crecimiento del hongo, se procedió a la identificación del agente causal por métodos fenotípicos o moleculares (espectrometría de masas o secuenciación de genes específicos). Los datos se consignaron en una ficha epidemiológica en formato Excel^®^.

### Identificación

El aislamiento obtenido en los medios de cultivo, incubados a 28 y 37 °C, se identificó fenotípicamente por observación microscópica del microcultivo con montaje húmedo con azul de lactofenol. La identificación molecular se realizó por espectrometría de masas (MALDI-TOF, VITEK MS® o Bruker®) y por secuenciación de los genes *ITS1*-*5.8S*-*ITS2* y calmodulina.

Se hizo la georreferenciación de todos los casos clínicos. Las imágenes fueron creadas con Adobe Illustrator®.

## Resultados

En el período estudiado se diagnosticaron 54 casos de esporotricosis. La mediana de edad de los pacientes fue de 35 años (rango: 11 a 69 años). Treinta y nueve pacientes fueron hombres, 14 mujeres y uno sin dato. En el cuadro 1 se muestran otros datos demográficos.

**Cuadro 1.** Datos epidemiológicos

Lugar de residencia*	n	Ocupación	n
Buenos Aires**	9	Veterinario	4
Corrientes	14	Tarea rural	19
Salta	3	Trabajo manual	3
Paraguay	1	Empleados	8
Santa Fe	5	Salud	1
Misiones	2	Otras	8
Santiago del Estero	1	Sin datos	11
Tucumán	2		
Chaco	2		
San Luis	1		
Santa Cruz	8		
Sin datos	6		

* Al momento de la infección

** Ciudad Autónoma de Buenos Aires, Provincia de Buenos Aires

La descripción de las formas clínicas, la localización de las lesiones y la probable fuente de infección y las comorbilidades se presentan en el cuadro 2. La forma clínica más frecuente fue la linfangítica nodular (54 %), seguida por la forma cutánea fija (32 %). La localización en miembros superiores (63 %) fue la preponderante en esta serie de casos.

**Cuadro 2:** Datos clínicos

Localización de las lesiones	n
Miembros superiores	34
Miembros inferiores	11
Otra localización cutánea	3
Pulmones	3
Diseminada	2
Sin dato	1
Forma clínica	n
Fija	17
Linfangítica	29
Pulmonar	3
Articular	1
Diseminada	4
Fuente probable de infección	n
Traumatismos con vegetales	10
Traumatismo	16
Arañazo de gato	17
Caza de mulitas	2
Inhalatoria	3
Desconocida	5
Comorbilidades	n
Ninguna	18
Diabetes	3
Hipotiroidismo	2
Infección con micobacterias (*Mycobacterium tuberculosis* y otras)	3
VIH	3
COVID	1
Hipertensión arterial	4
Linfoma	1
Embarazo	1
EPOC	1
Histoplasmosis	1
Cirugía cardíaca previa	1

Las formas diseminadas tuvieron compromiso cutáneo, ganglionar, visceral con hepatoesplenomegalia y uno de los casos presentó infiltrado pulmonar cavitado. En solo un caso se pudo comprobar la inmunosupresión del paciente por infección con HIV. Por otra parte, las formas pulmonares se presentaron con fiebre, tos y expectoración mucopurulenta. Uno de los casos era una paciente fumadora, hipertensa y cardiópata; otro caso, un paciente diabético, y el tercero mostró compromiso pleural.

**Figura 1:**
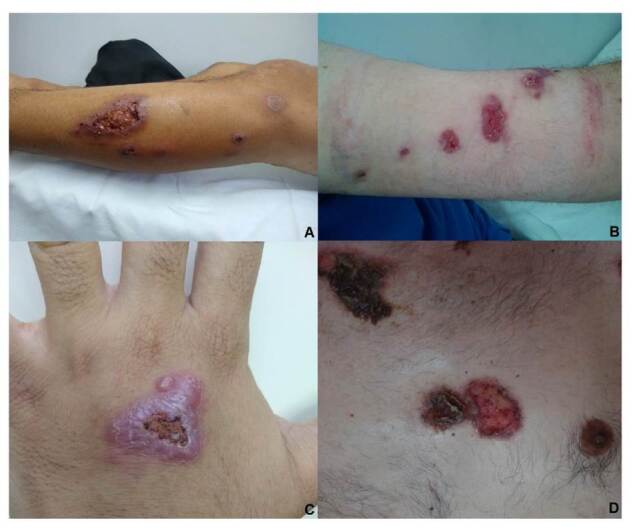
A y B. Lesión en miembro inferior izquierdo y en antebrazo izquierdo de esporotricosis linfangítica nodular. C. Lesión en mano de esporotricosis cutánea fija. D. Lesiones en tronco de esporotricosis diseminada.

**Figura 2:**
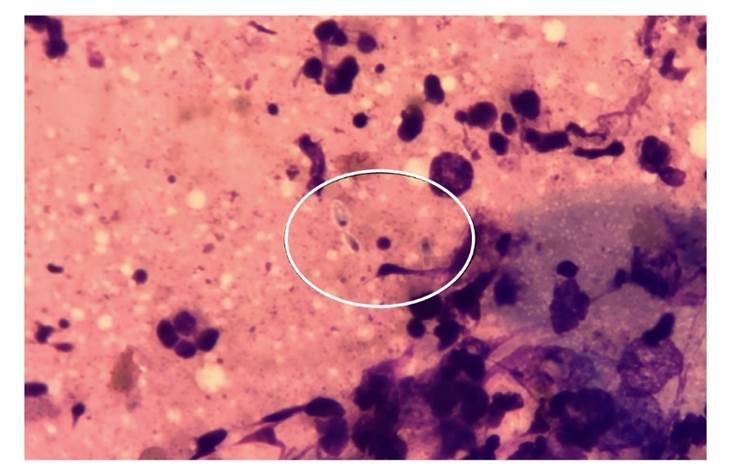
Lesión de pierna del paciente de la figura 1A. Giemsa, 1.000X

**Figura 3:**
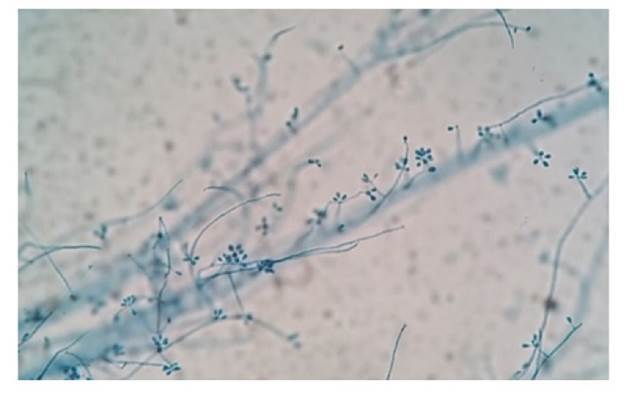
Microcultivo a 28 °C de *Sporothrix* spp., obtenido en cultivo primario. Azul de lactofenol, 400X.

La figura 1 muestra lesiones de diferentes formas clínicas. En más del 85 % de los casos, la punción o biopsia del nódulo subcutáneo fue la muestra que permitió el diagnóstico (cuadro 3). Todos los pacientes fueron diagnosticados por medio del cultivo positivo, mientras que el rendimiento del examen directo fue del 19,2 %.En la figura 2 se muestra la coloración de Giemsa de la muestra clínica obtenida por escarificación de la lesión cutánea de la pierna del paciente de la figura 1A. La imagen del microcultivo de *Sporothrix* spp. se presenta en la figura 3.

**Cuadro 3:** Muestras que posibilitaron el diagnóstico y las especies aisladas

Muestras diagnósticas	n
Punción o biopsia de nódulo subcutáneo	46
Escarificación cutánea	5
Lavado broncoalveolar	2
Líquido sinovial	1
Especies aisladas	n
*Sporothrix schenckii* complex	32
*Sporothrix schenckii sensu stricto*	12
*Sporothrix brasiliensis*	9
*Sporothrix globosa*	1

Las especies aisladas se listan en el cuadro 3. Cabe destacar que solamente en 22 casos se realizó la identificación a nivel de especie mediante técnicas moleculares. En los otros 32 casos se identificó como *S. schenckii sensu lato*.

*Sporothrix brasiliensis* se aisló en 8 de 9 casos de pacientes que habían sido arañados o mordidos por gatos; *S. globosa* se encontró en un enfermo que había sufrido traumatismos con árboles. En dos casos en los que se aisló *S. schenckii sensu stricto*, la fuente de infección fueron los gatos, así como otros siete casos donde el agente etiológico fue identificado como complejo *S. schenckii* ya que no se realizó la identificación molecular.

**Figura 4:**
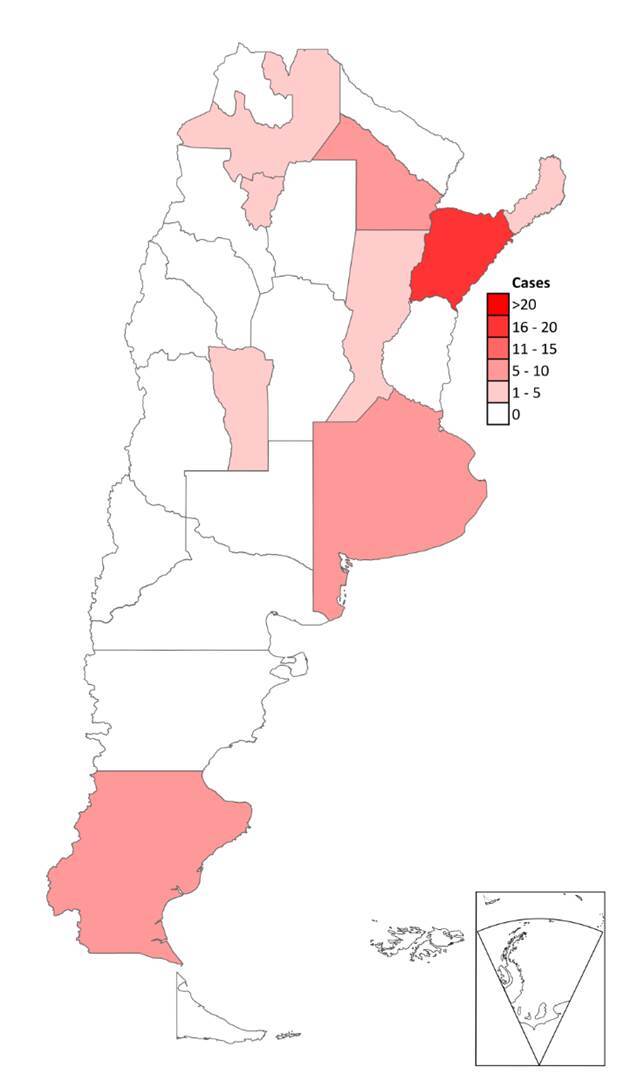
Georreferenciación de los casos presentados durante el período 2010-2022

La distribución geográfica de los casos presentados se muestra en la figura 4.

Un paciente no hizo el tratamiento y en 24 no se informó la recepción de la medicación. Los medicamentos utilizados y la duración del tratamiento se describen en el cuadro 4.

**Cuadro 4.** Esquemas terapéuticos utilizados

Tratamiento	n
Itraconazol	20
Itraconazol más termoterapia	1
Itraconazol más terbinafina	1
Anfotericina B	4
Anfotericina B más itraconazol	2
Crioterapia	1
Desconocido	24
Sin tratamiento	1
**Duración del tratamiento (meses)**	n
Hasta 2	3
4-6	5
9-12	6
Desconocido	39

## Discusión

La esporotricosis es la micosis de implantación de mayor prevalencia y distribución a nivel mundial ^2^^,^^3^^,^^7^. Se han descrito áreas hiperendémicas en Latinoamérica, Asia y África, que deben tenerse en cuenta sobre todo en pacientes que refieran viajes recientes ^3^^,^^10^. Aunque está estrechamente relacionada con personas que realizan tareas con alto riesgo de traumatismo, sobre todo agricultores, desde hace unos años comenzó a observarse su transmisión por animales como gatos domésticos y armadillos ^2^^,^^7^.

Esta enfermedad históricamente fue vinculada con una sola especie, *S. schenckii.* Actualmente, dentro de este género se reconocen varios clados que pueden vincularse con un área geográfica determinada, aunque aún en muchas regiones, la mayoría de los aislamientos no han sido estudiados en detalle ^7^. En relación con lo señalado, podemos decir que *S. schenckii sensu stricto* tiene una distribución geográfica amplia o global, *S. brasiliensis* es hiperendémica en Brasil y ha comenzado a diseminarse en otros países latinoamericanos como Paraguay, Argentina y Panamá ^2^. *Sporothrix globosa,* aunque tiene menor virulencia que las antes mencionadas ^14^, constituye el 99 % de todas las especies aisladas en Asia ^15^ y que ha sido descrita en diversas partes del mundo ^13^^,^^14^. *Sporothrix luriei* o *S. mexicana* fueron reportadas en pocas ocasiones ^2^^,^^7^.

En nuestra casuística se registraron casos en diferentes áreas geográficas de nuestro país. También se hizo el diagnóstico de esta micosis en un paciente que refirió haber adquirido la infección durante un viaje a Rio de Janeiro (Brasil). Estas regiones tienen diferentes características climáticas, de suelo y tipo de vegetación. Esta distribución se puede observar en la figura 4.

En Rio de Janeiro, donde la población de gatos callejeros es elevada, se han detectado más de 5.000 casos en los últimos años. Estos animales son los protagonistas de la transmisión de la esporotricosis al hombre en un brote zoonótico sin precedentes ^3^^,^^4^. La especie responsable de esta zoonosis es *S. brasiliensis*^11^. Recientemente, fue publicado un trabajo que relaciona la alta carga fúngica en las gotitas respiratorias expulsadas en el estornudo de los gatos infectados con la esporotricosis en humanos ^5^ y, en especial, con las formas extracutáneas como la granulomatosa conjuntival ^2^.

El dimorfismo térmico de *Sporothrix* es una adaptación morfológica importante que condiciona la patogenicidad de esta infección y es compartida con otros agentes patógenos humanos, filogenéticamente distantes, como aquellos de los órdenes Onygenales y Eurotiales. Las especies de *Sporothrix* anidadas en el clado patogénico son hongos termodimorfos que responden de manera más eficiente a los estímulos térmicos. *Sporothrix brasiliensis* expresa diferentes mecanismos de virulencia como termotolerancia y expresión de adhesinas y melanina, y es la especie más virulenta en algunos modelos de ratón. Esta especie se asocia con formas atípicas y más graves de la enfermedad, incluida la infección cutánea diseminada en huéspedes inmunocompetentes y la enfermedad sistémica ^2^^,^^3^. Asimismo, parece ser menos sensible a determinados agentes antifúngicos como el itraconazol o la anfotericina B ^10^.

Otras especies como *S*. *schenckii*, *S. globosa* o *S. luriei* son responsables de un número mucho menor de casos de esta enfermedad en estas latitudes. En otros estados brasileños, como el de Rio Grande do Sul, los casos de esporotricosis también están aumentando, aunque en menor medida ^16^.

En los casos de nuestro país, de los pacientes que sufrieron heridas o inoculación por el contacto con gatos, se aisló *S. brasiliensis* en 8 de 17 casos y *S. schenckii sensu stricto* en dos casos; en los siete casos restantes no se hizo la identificación molecular. Por otra parte, *S. globosa* se asoció a traumatismos con elementos vegetales, aunque cinco de los casos en los que se identificó *S. schenckii sensu stricto* como agente causal también tuvieron relación con el traumatismo con material vegetal.

En todos los casos presentados en este trabajo, el diagnóstico se realizó por los métodos convencionales a partir del examen micológico de las diferentes muestras clínicas. La punción o biopsia de nódulos subcutáneos fue la muestra clínica con mejor rendimiento para el diagnóstico (85 % de los casos). El cultivo en medios habituales y a dos temperaturas sigue siendo el método para la confirmación del diagnóstico frente al examen directo en fresco o coloreado, los cuales tuvieron un escaso 19,2 % de positividad.

El avance de esta micosis en ciertas áreas geográficas y la presencia de *Sporothrix* spp. en los gatos generó la investigación y la aplicación de técnicas rápidas para el diagnóstico de esta micosis ^17^. En un estudio llevado a cabo en Brasil, se realizó la detección de anticuerpos anti-*Sporothrix* por la técnica de inmunocromatografía en sueros de 100 pacientes con diferentes formas clínicas. Esta técnica mostró una sensibilidad del 83 % y una especificidad del 82 %. Estos resultados evidenciaron que Anti-Sporo LFA, IMMY® es una herramienta prometedora para el diagnóstico rápido de esta micosis ^17^.

El tratamiento de elección en las formas cutáneas o linfocutáneas es el itraconazol a una dosis diaria de 6 a 10 mg/kg con un máximo de 400 mg por día ^12^^,^^18^. Solo en México se utiliza el yoduro de potasio como primera alternativa ^12^^,^^19^. En las formas diseminadas o extracutáneas, el tratamiento se lleva a cabo inicialmente con anfotericina B y luego se continúa con itraconazol ^6^.

El tiempo promedio de tratamiento es de 3 a 6 meses y siempre se recomienda continuar 2 a 3 semanas más, una vez que se observa mejoría clínica ^16^.

En los últimos años se ha observado una pobre respuesta al tratamiento con itraconazol en especial con los casos de *S. brasiliensis*^20^. Aunque aún no hay una recomendación general, en algunos casos se podría utilizar terapia combinada con terbinafina o yoduro de potasio ^19^^,^^21^. La respuesta terapéutica no solo está ligada a la especie implicada, sino también a la forma clínica y las comorbilidades del huésped ^16^.

En esta serie de casos el itraconazol fue la medicación más utilizada, aunque en tres casos se indicó la terapia combinada por la escasa respuesta clínica con la monoterapia. Esta parece ser una alternativa cuando la respuesta no es la esperada.

En Argentina, la esporotricosis linfangítica nodular es la forma clínica más frecuente. Es importante destacar la presencia de *S. brasiliensis* y su transmisión zoonótica ya que generó la aparición de casos en zonas con clima poco favorable para esta micosis. Con lo anterior, el reconocimiento de esta infección es necesaria porque puede acontecer en regiones no habituales. De igual forma, se requiere impulsar la identificación molecular de los aislamientos para conocer la distribución geográfica de las especies de este género.

## References

[1] Lopes-Bezerra LM, Mora-Montes HM, Zhang Y, Nino-Vega G, Rodrigues AM, de Camargo ZP (2018). Sporotrichosis between 1898 and 2017: The evolution of knowledge on a changeable disease and on emerging etiological agents. Med Mycol.

[2] Rossow JA, Queiroz-Telles F, Caceres DH, Beer KD, Jackson BR, Pereira JG (2020). A One health approach to combatting Sporothrix brasiliensis: narrative review of an emerging zoonotic fungal pathogen in South America. J Fungi (Basel).

[3] Rodrigues AM, Della Terra PP, Gremião ID, Pereira SA, Orofino-Costa R, de Camargo ZP. (2020). The threat of emerging and re-emerging pathogenic Sporothrix species. Mycopathologia.

[4] Cabañes FJ. (2020). Esporotricosis en Brasil: animales + humanos = una sola salud. Rev Iberoam Micol.

[5] de Andrade Galliano Daros Bastos F, Raimundo Cognialli RC, Rodrigues de Farias M, dos Santos Monti F, Wu K, Queiroz-Telles F. (2022). Spread of Sporothrix spp. through respiratory droplets from infected cats: A potential route of transmission. Med Mycol.

[6] Bonifaz A, Tirado-Sánchez A. (2017). Cutaneous disseminated and extracutaneous sporotrichosis: Current status of a complex disease. J Fungi.

[7] Chakrabarti A, Bonifaz A, Gutierrez-Galhardo MC, Mochizuki T, Li S. (2015). Global epidemiology of sporotrichosis. Med Mycol.

[8] Han HS, Kano R. (2020). Feline sporotrichosis in Asia. Braz J Microbiol.

[9] Martínez Cepeda GE. (2016). Esporotricosis en caninos y felinos: hallazgos clínicos, métodos de diagnóstico y tratamiento. Analecta Vet.

[10] Rodrigues AM, Gonçalves SS, de Carvalho JA, Borba-Santos LP, Rozental S, Camargo ZP. (2022). Current progress on epidemiology, diagnosis, and treatment of sporotrichosis and their future trends. J Fungi.

[11] Gremião ID, Miranda LH, Pereira-Oliveira GR, Menezes RC, Machado AC, Rodrigues AM (2022). Advances and challenges in the management of feline sporotrichosis. Rev Iberoam Micol.

[12] Queiroz-Telles F, Bonifaz A, Cognialli R, Lustosa BPR, Vicente VA, Ramírez-Marín HA. (2022). Sporotrichosis in children: Case series and narrative review. Curr Fungal Infect Rep.

[13] Mora-Montes HM. (2022). Sporothrix and sporotrichosis 2.0. J Fungi.

[14] Nava-Pérez N, Neri-García LG, Romero-González OE, Terrones-Cruz JA, García-Carnero LC, Mora-Montes HM. (2022). Biological and clinical attributes of Sporothrix globosa, a causative agent of sporotrichosis. Infect Drug Resist.

[15] Rudramurthy SM, Shankarnarayan SA, Hemashetter BM, Verma S, Chauhan S, Nath R (2021). Phenotypic and molecular characterisation of Sporothrix globosa of diverse origin from India. Braz J Microbiol.

[16] Poester VR, Basso RP, Stevens DA, Munhoz LS, de Souza Rabello VB, Almeida-Paes R (2022). Treatment of human sporotrichosis caused by Sporothrix brasiliensis. J Fungi.

[17] Cognialli R, Bloss K, Weiss I, Caceres DH, Davis R, Queiroz-Telles F. (2022). A lateral flow assay for the immunodiagnosis of human cat-transmitted sporotrichosis. Mycoses.

[18] Kauffman C.A., Bustamante B., Chapman S.W., Pappas P.G. (2007). Clinical practice guidelines for the management of sporotrichosis: 2007 update by the Infectious Diseases Society of America. Clin Infect Dis.

[19] Bonifaz A, Vázquez-González D. (2013). Diagnosis, and treatment of lymphocutaneous sporotrichosis: What are the options?. Curr Fungal Infect Rep.

[20] Almeida-Paes R, Oliveira MME, Freitas DFS, Valle ACFD, Gutierrez‑Galhardo MC, Zancopé-Oliveira RM. (2017). Refractory sporotrichosis due to Sporothrix brasiliensis in humans appears to be unrelated to in vivo resistance. Med Mycol.

[21] Zhang X, Huang H, Feng P, Zhang J, Zhong Y, Xue R (2011). In vitro activity of itraconazole in combination with terbinafine against clinical strains of itraconazole-insensitive Sporothrix schenckii. Eur J Dermatol.

